# Reconsidering the Interpretation of “Recurrence-Free Survival” After Mastectomy for DCIS. Comment on Sae-sim et al. Tamoxifen Reduces Breast Cancer Recurrence in Women with DCIS Who Underwent Mastectomy. *Curr. Oncol.* 2026, *33*, 89

**DOI:** 10.3390/curroncol33040223

**Published:** 2026-04-17

**Authors:** Janhavi Venkataraman, Kefah Mokbel

**Affiliations:** The London Breast Institute, Princess Grace Hospital, HCA Healthcare Private Limited, London W1U 5NY, UK

Sae-sim et al. report a substantial reduction in recurrence among women receiving adjuvant tamoxifen after mastectomy for ductal carcinoma in situ (DCIS), with a hazard ratio (HR) of 0.178 (95% CI 0.061–0.516; *p* = 0.001) and a 10-year recurrence-free survival (RFS) of 94.7% in the endocrine therapy (ET) group versus 77.9% in the non-ET group [1]. This corresponds to an absolute difference of 16.8 percentage points and an implied 82% relative risk reduction.

At the outset, the Abstract reports a 10-year RFS of 68.7% for the non-ET group, contradicting both the Results section and the Kaplan–Meier curve (77.9%). Because the magnitude of effect depends directly on the control-arm estimate, this discrepancy requires formal correction and clarification of the denominators and event counts underpinning the reported hazard ratio and survival estimates.

More fundamentally, the interpretation of the primary endpoint warrants reconsideration. Of the 16 total events reported, 11 (68.8%) occurred in the contralateral breast. After mastectomy, contralateral cancers represent new primary tumours rather than a recurrence of the index DCIS. Excluding contralateral events, only five events remain across the entire cohort (5/180; approximately 2.8%). This crude rate aligns closely with established long-term ipsilateral recurrence rates following mastectomy for pure DCIS. A meta-analysis reported local recurrence rates of 1.6% with negative margins and 5.3% with close or positive margins [2]. A large systematic review including 9404 patients demonstrated an adjusted 10-year ipsilateral recurrence rate of 2.6% (95% CI 0.8–4.5%) [3]. Thus, when contralateral events are excluded, the observed recurrence rate does not appear materially higher than that reported in the literature.

These observations suggest that the composite endpoint analysed is dominated by contralateral new primaries rather than true post-mastectomy recurrence. In this context, the term “recurrence-free survival” may be conceptually misleading. A more accurate descriptor would be subsequent breast cancer-free survival, reflecting the inclusion of both ipsilateral and contralateral events. This distinction is not semantic; it directly affects causal interpretation. Tamoxifen’s preventive effect on contralateral breast cancer is well established. The TAM-01 randomised trial demonstrated a significant reduction in contralateral breast cancer incidence with low-dose tamoxifen (HR 0.36; 95% CI 0.14–0.92; *p* = 0.025) [4]. Without stratified reporting of ipsilateral and contralateral events by treatment arm, it is not possible to determine whether the observed HR of 0.178 reflects improved local control after mastectomy or primarily captures a known chemopreventive effect.

An additional unresolved issue is whether the contralateral event rate itself exceeds that expected in comparable DCIS populations. The manuscript does not provide contextual benchmarking to allow this assessment.

The reported hazard ratio is derived from only 16 total events and is accompanied by a wide confidence interval (0.061–0.516), indicating statistical fragility. In a non-randomised design with physician-directed treatment allocation, such an effect size is highly susceptible to residual confounding. The multivariable Cox model did not include comedonecrosis despite a statistically significant imbalance between groups (36.7% vs. 22.5%; *p* = 0.044) and its recognised prognostic relevance. Given the limited number of events, model stability is inherently constrained.

Follow-up duration also differed significantly between groups (8.52 vs. 5.94 years; *p* < 0.001), yet no sensitivity analyses—such as landmark analysis or restricted mean survival time—were reported. Three breast cancer–related deaths occurred during follow-up. In small cohorts, standard Kaplan–Meier methods may overestimate event-free survival when competing risks are present; competing-risk regression (e.g., Fine–Gray modelling) would provide more appropriate estimates.

The median time to event was 1.93 years, with several events occurring within approximately one year of surgery. Very early post-mastectomy events may represent occult invasive or residual disease present at baseline rather than true DCIS recurrence. Sensitivity analyses excluding early events were not presented.

Taken together, the inconsistency in reported survival estimates, the reliance on a composite endpoint dominated by contralateral primaries, the small number of events underpinning the hazard ratio, the imbalance in follow-up duration, and limited adjustment for confounding substantially limit causal inference. Clear separation of ipsilateral recurrence from contralateral new primary events, and precise definition of the analysed endpoint, are essential before concluding that tamoxifen confers a mastectomy-specific reduction in recurrence risk.

Given the absence of demonstrated overall survival benefit with tamoxifen in this setting [5], together with its established adverse effects—including thromboembolic complications and endometrial carcinoma—accurate quantification of benefit in a non-standard post-mastectomy context is essential to support informed clinical decision-making.

Notably, NCCN guidelines do not include a uniform consensus recommendation for adjuvant endocrine therapy in patients with DCIS treated with mastectomy; panel members have explicitly acknowledged that tamoxifen use in this setting remains discretionary [6]. This absence of guideline consensus makes the accurate and reliable quantification of benefit from observational data particularly important before any change in clinical practice is considered.

We respectfully request the correction of the Abstract to ensure concordance with the Results section and Kaplan–Meier figure, and clarification of endpoint definitions, event distribution by laterality and treatment arm, and analytic methods used to derive the reported hazard ratio.
